# *Wolbachia* wSinvictaA Infections in Natural Populations of the Fire Ant *Solenopsis invicta*: Testing for Phenotypic Effects

**DOI:** 10.1673/031.011.0111

**Published:** 2011-02-04

**Authors:** Andrew M. Bouwma, DeWayne Shoemaker

**Affiliations:** ^1^ Department of Entomology and Nematology, University of Florida, Bldg. 970 Natural Area Drive, Gainesville, FL 32611, USA.; ^2^ USDA-ARS, Center for Medical, Agricultural, and Veterinary Entomology, Gainesville, FL, 32608, USA

**Keywords:** cytoplasmic incompatibility, fitness, social insects, *Solenopsis invicta*, *Wolbachia*

## Abstract

*Wolbachia* are intracellular bacteria that commonly infect many arthropods and some nematodes. In arthropods, these maternally transmitted bacteria often induce a variety of phenotypic effects to enhance their own spread within host populations. *Wolbachia* phenotypic effects generally either provide benefits to infected host females (cytoplasmic incompatibility, positive fitness effects) or bias host sex ratio in favor of females (male-killing, parthenogenesis, feminization), all of which increase the relative production of infected females in host populations. *Wolbachia* surveys have found infections to be exceedingly common in ants, but little is known at this juncture as to what phenotypic effects, if any, they induce in this group. Previous studies have demonstrated that individuals from native populations of the invasive fire ant *Solenopsis invicta* commonly harbor one or more of three *Wolbachia* variants. One of the variants, wSinvictaA, typically occurs at low prevalence in *S. invicta* populations, appears to have been transmitted horizontally into *S. invicta* three or more times, and has been lost repeatedly from host lineages over time. In order to determine the phenotypic effects and likely population dynamics of wSinvictaA infections in these ants, brood production patterns of newly mated fire ant queens were studied during simulated claustral founding and measured wSinvictaA transmission fidelity within mature single-queen families. No clear evidence was found for *Wolbachia-mduced* cytoplasmic incompatibility, significant fitness effects, or male-killing. Maternal transmission was perfect to both virgin queens and males. Possible mechanisms for how this variant could be maintained in host populations are discussed.

## Introduction

*Wolbachia* are endosymbiotic bacteria found in a wide range of arthropods and filarial nematodes (Bandi et al. 1998; O'Neill et al. 1992, 1995). Since these microbes are primarily transmitted via the egg cytoplasm, their fitness is tightly linked to host reproduction and generally should be selected to increase host fitness (Lipsitch et al. 1996). However, since host males are a ‘dead end’ for symbiont transmission, numerous *Wolbachia* variants have evolved mechanisms to manipulate host reproduction in ways that provide benefits to infected host females, despite often decreasing the fitness of infected host males. For example, male-killing (MK) Wolbachia kill the males they infect that could result in diverting limited resources to infected females, and Wolbachia that induce cytoplasmic incompatibility (CI) modify the sperm such that infected males are incompatible with uninfected females effectively sterilizing both when such matings occur (Hoffmann and Turelli 1997). Depending on the strength of CI and the prevalence of infected males in the population, CI can provide a strong mating advantage to infected females, since infected females can mate with either class of males. Cytoplasmic incompatibility (and sex-ratio distorting phenotypic effects such as MK, feminization of genetic males, thelytokous parthenogenesis) induced by *Wolbachia* increase the production of infected females relative to uninfected females in populations, enabling infections to spread. While *Wolbachia* variants are favored that increase the fitness of host females, theoretical models and empirical studies show that variants with direct negative fitness effects also can spread in host populations (Hoffmann and Turelli 1997; Hurst et al. 1997; Turelli 1994; Weeks et al. 2007; Werren 1997).

CI is one of the most common phenotypic effects induced by *Wolbachia* infections in arthropods (Charlat et al. 2001; Hoffmann and Turelli 1997). A large body of theoretical and empirical work has identified several parameters for CI-inducing *Wolbachia* that can be used as clues in understanding the population dynamics of infections in host populations: (I) the strength of CI (proportion of embryos from crosses of infected males and uninfected females that do not hatch), (II) the fidelity of maternal transmission, (III) the effects of infections on host female fitness, and (IV) the prevalence of infection (proportion of infected individuals in a population). Strong CI, high efficiency of maternal transmission, and low fitness costs can enable a *Wolbachia* variant to spread quickly and reach high equilibrium prevalences in host populations (Hoffmann and Turelli 1997). Moreover, theoretical models predict that the stable equilibrium prevalence of a CI- inducing *Wolbachia* cannot be below 50% (Hoffmann and Turelli 1997). *Wolbachia* variants also can be maintained in host populations in the absence of CI (or sex-ratio-distortion) (Charlat et al. 2004; Hoffmann et al. 1996) if they induce positive fitness effects, or, in the absence of fitness effects, if they have perfect maternal transmission or are frequently transmitted horizontally (Hoffmann and Turelli 1997).

Previous studies of *Wolbachia* in the fire ant *Solenopsis invicta* in South America (Ahrens and Shoemaker 2005; Shoemaker et al. 2003; Shoemaker et al. 2003; Shoemaker et al. 2000) have revealed that the evolutionary histories and population dynamics of *Wolbachia* infections in these ants are complex, but have not provided clear evidence for any one particular phenotypic effect. Shoemaker et al. (2003) found that *Wolbachia* were not associated with decreased mtDNA diversity in *S*. *invicta*, a pattern expected if a *Wolbachia* variant has recently swept through populations and replaced all other mtDNA haplotypes with its associated mtDNA. Ahrens and Shoemaker (2005) found that infected populations comprised one or more of three different variants (wSinvictaA, wSrichteriA, and wSinvictaB) based on the highly variable *Wolbachia* surface protein (wsp) gene, and the prevalence of each of these variants in six of eight infected populations was well below the 50% minimum stable equilibrium prevalence predicted for CI-inducing *Wolbachia* by theoretical models (Hoffmann and Turelli 1997). The most widespread variant, wSinvictaA, never exceeded a prevalence of 28% in the five geographically divergent populations where it was found (Ahrens and Shoemaker 2005). The maternal transmission fidelity of this variant remains unknown since previous estimates reported by Shoemaker et al. (2000) comprised data mostly from wSinvictaB-infected families. Ahrens and Shoemaker (2005) also mapped the distribution of these three *Wolbachia* variants onto a phylogeny of host mtDNA sequences, and concluded that the history of *Wolbachia* infections in *S. invicta* is ancient and has involved multiple, independent invasions or horizontal transmission events coupled with secondary loss within different host (mtDNA) lineages.

Based on these combined results, at least four alternative scenarios could explain the apparent instability of wSinvictaA infections in host lineages and the low infection prevalence observed in fire ant populations. These four scenarios include: I) the wSinvictaA variant induces CI, but combined negative fitness effects and imperfect transmission prevent it from reaching a stable equilibrium prevalence; II) wSinvictaA variant is a sex-ratio distorting *Wolbachia* in this ant (e.g. male-killers), which frequently have much lower equilibrium prevalence and high rates of loss of infection (Hurst et al. 1997); III) wSinvictaA induces small fitness benefits, but persists at low prevalence because of inefficient maternal transmission; IV) or the wSinvictaA *Wolbachia* variant does not induce any phenotypic (or fitness) effects in *S. invicta* to promote its spread and persists at low prevalence because of near-perfect maternal transmission coupled with horizontal transmission.

The objective of the present study was to identify what phenotypic effects, if any, the *Wolbachia* variant wSinvictaA induces in its natural host *S. invicta.* Understanding the effects this *Wolbachia* variant induces in *S. invicta* potentially could shed light on the apparent low prevalence of infections within some populations described above as well as provide relevant baseline information for evaluating the potential for using *Wolbachia* for fire ant biological control. To accomplish this objective, *Wolbachia-*infected and uninfected newly mated *S. invicta* queens from mating flights in Argentina were collected and queen productivity and brood production patterns in the laboratory were examined during simulated colony founding for evidence of CI or fitness effects. In addition, *Wolbachia* prevalence in host populations and transmission fidelity to male and female reproductive offspring in mature colonies in the field were measured. As shown below, the results in combination with those from previous studies provide one of the most detailed pictures yet of the relationship between a *Wolbachia* variant and its ant host.

## Materials and Methods

### Study area

A prerequisite for the proposed study was identification of a population with intermediate prevalence of *Wolbachia* infections, where all infected individuals harbor the same *Wolbachia* variant. Intermediate prevalence of *Wolbachia* infections increases the probability of obtaining sufficient sample sizes of both infected and uninfected ants as well as increasing the efficiency of the experiments (infection status can only be determined post hoc and controlled laboratory crosses for measuring offspring production are not possible). The presence of multiple *Wolbachia* variants would confound fitness assays, since some infected individuals could suffer reduced fitness due to bidirectional CI (Werren 1998). Also a study site with only the single-queen (monogyne) social form was preferred since fecundity and weight differences existing between queens of the two social forms (DeHeer 2002) would have confounded tests for effects of *Wolbachia* infection. To identify a collecting site meeting the above criteria, samples of ∼20 workers (including sexuals when available) were collected from 44–86 *S. invicta* colonies at each of ten sites throughout northern Argentina in May and June 2004. All collected ants were placed into vials containing 95% ethanol in the field and subsequently stored at -20° C pending DNA analyses. Subsequent analyses of these samples utilizing the *Wolbachia* and *Gp-9* assays (see below) revealed that *S. invicta* populations in the vicinity of the cities of Eldorado and Puerto Iguazú (Misiones province) in northeastern Argentina consisted exclusively of monogyne colonies with an intermediate prevalence of a single *Wolbachia* variant (wSinvictaA; Table 1). Thus, collecting efforts were focused on this region for the present study.

### Queen protocols

Putative newly mated *S. invicta* queens were collected from the ground during two larges-cale mating flights in Puerto Iguazú on 1 (cohort 1; *N* = 199) and 28 (cohort 2; *N* = 99) November 2004. Collecting sites for the two cohorts were separated by less than 1 km and were located within the city limits of Puerto Iguazú. Collected queens were kept in ventilated plastic cups (with plaster bottoms, moistened with water) in groups of approximately 20 individuals at 4° C while in Puerto Iguazú and then in an ice chest while *en route* to a laboratory at the Facultad de Ciencias Exactas Químicas y Naturales of the Universidad Nacional de Misiones (UNM) in Posadas, Argentina.

All queens were weighed to the nearest 0.1 mg and placed into individual rearing tubes with a water supply within 72 hours (cohort 1) or 48 hours (cohort 2) after collection as described by Tschinkel (1993). Queens were maintained in darkness (except during observation) and at ambient temperature (cohort 1 = 21–31° C, cohort 2 = 25–31° C) and humidity (cohort 1 = 40–80%; cohort 2 = 41–74%). These conditions closely mimic those experienced by queens during the claustral colony founding stage of *S. invicta*, during which newly mated queens seal themselves off in burrows and rear the first generation of female worker offspring on trophic eggs produced from their own fat stores (Tschinkel 1993).

Queens were checked daily for survival, and brood production patterns suggestive of CI or diploid male production were noted. While direct evidence for CI has not yet been found in ants, two different CI phenotypes have been observed in other haplodiploids: (I) production of males from all or a proportion of fertilized eggs (male development, MD; Breeuwer and Werren 1990), and (II) the death of all or some proportion of female embryos (female mortality, FM; Vavre et al. 2000). An intermediate phenotype, for which CI results in some fertilized eggs dying and (from the same infected mothers) some fertilized eggs developing into males, was observed in artificially developed laboratory lines of the parasitoid wasp, *Leptopilina heterotoma,* by Vavre et al. (2001).

While no sexual brood (males or alate females) typically is produced during colony founding, some *S. invicta* queens produce diploid male offspring as a result of mating with a male with an identical allele at the sex locus (Ross and Fletcher 1986). Consequently, the brood production patterns of such diploidmale-producing (DMP) queens are similar to those expected for MD CI An intermediate CI phenotype, such as that observed by Vavre et al. (2001) would appear similar to MD CI or DMP due to the presence of male offspring.

Complete FM CI is easily recognized as a complete failure to produce viable offspring, while incomplete FM CI is more cryptic, causing a reduction in offspring production. Strong incomplete FM CI should be recognized by the presence of bimodality in frequency distributions of brood productivity, with expressers clustering at very low productivity. Brood production patterns of queens were evaluated and classified to a particular brood production phenotype only if they survived until the end of the study, which reduced the likelihood that individuals classified with putative CI were afflicted with some other ailment that could have interfered with their brood production.

For cohort 1, observations were continued until either the death of the queen or the day the first workers eclosed. Each nest was dismantled on the day the first workers eclosed, or at the end of the study (day 45) if the colony did not produce viable worker brood. For cohort 2, the study was terminated before the eclosion of workers (18 total days of observation). On the final collection day for each colony in both cohorts, all worker adults, pupae, and larvae present were counted within the nest unit and weighed each queen to the nearest 0.1 mg. All material was then stored in vials containing 95% ethanol for subsequent morphometric and genetic analyses. For morphometric analysis, the head of each queen was photographed through a dissecting microscope with a digital camera, and subsequently measured head width from the image using the measurement tool in Adobe Photoshop (2002). For queens that did not produce any viable female offspring, the gaster was dissected and the spermatheca was examined under magnification to ensure that they were mated. All statistical analyses were conducted using JMP 5.0.1.2 or SAS 8.2.

### Genomic DNA extractions

All genomic DNA extractions were performed using the Puregene DNA isolation kit (Gentra Systems) following Shoemaker et al. (2000). For the initial survey aimed at identifying a suitable collecting site for the queen studies, DNA was bulk extracted from five workers per colony to reduce the likelihood of missing *Wolbachia* infections within colonies or “b”like alleles at *Gp-9* present in most, but not all, workers of polygyne colonies (see below). For all queens collected from mating flights and subsequently used for the laboratory studies below, *Wolbachia* infection status and social form were determined post hoc using the head as the source of material for DNA extractions (Shoemaker et al. 2000).

To estimate the maternal transmission fidelity of the *Wolbachia* variant wSinvictaA, DNA was extracted separately from virgin alate females from mature colonies harboring this variant that were identified in the initial survey. Finally, DNA was also extracted from individual males from mature colonies with the wSinvictaA variant to determine whether males are infected and, thus survive infection.

### Screening for *Wolbachia* and Microsporidia

Extracted genomic DNA samples were surveyed for the presence of *Wolbachia* using a PCR assay incorporating the primers *Wsp*81F and *Wsp*691R (Zhou et al. 1998). Details of the PCR mixes, PCR profiles, and subsequent electrophoresis and visualization of PCR products are described in Shoemaker et al. (2003, 2000). The presence of *Wolbachia* in a subset of the queens (*N* = 147) was also screened for using the long PCR protocol detailed in Jeyaprakash and Hoy (2000). Total genomic DNA from all queens also was surveyed for the presence of two Microsporidia, *Kneallhazia solenopsae* and *Vairimorpha invictae,* using the multiplex PCR assay of Valles et al. (2004). The stage one *Gp-9* PCR (see below) was used as a control for assessing DNA template quality for all *Wolbachia* and microsporidia assays.

### Determination of social form

The *Gp-9* genotype of each queen was determined using a two-stage, allele-specific PCR assay. This assay is designed to amplify *b*-like alleles only, which occur only in the polygyne social form (see Krieger and Ross 2002; Ross et al. 2003). Queens that are negative for the stage-2 PCR are *BB* queens, and this includes all queens of the monogyne social form and some of the polygyne form. However, while some newly mated polygyne queens may be *BB* (DeHeer 2002), a complete absence of *b*-like alleles in surveys of newly mated queens, as was observed in initial population surveys of mature colonies at Puerto Iguazú, would make the presence of polygyne queens in this sample highly unlikely.

### Sequencing of *Wolbachia* variants

A portion of the *Wolbachia* surface protein (*wsp*) gene was sequenced from all *Wolbachia-*infected samples to identify the *Wolbachia* variant infecting each ant. *Wolbachia* DNA was PCR-amplified using the primers *Wsp*81F and *Wsp*691R. PCR reaction components, thermal cycling conditions, and sequencing methods were identical to those described in Ahrens and Shoemaker (2005).

## Results

A total of 298 putative newly mated queens were collected from two mating flights in Puerto Iguazú. Post hoc *Wolbachia* surveys and sequence analyses showed that 48 of these queens were infected with the single *Wolbachia* variant wSinvictaA, for a population prevalence of 0.161 (95% CI = 0.124, 0.207). Long PCR and standard PCR assays produced identical results. Eight individuals lacking *Wolbachia* harbored microsporidia infections (five infected with *Vairimorpha invictae,* three with *Kneallhazia solenopsae).* Since microsporidia infections can be debilitating in *S. invicta* (Oi and Williams 2002), these eight queens were excluded from all subsequent data analyses. Not surprisingly, since no evidence for polygyny was found in the initial survey of mature colonies in the Puerto Iguazú area (Table 1), none of the newly mated queens collected at the Puerto Iguazú site possessed *b*-like alleles at *Gp-9* characteristic of polygyny. Based on these results, it was concluded that polygyny was absent from the sample.

### Brood production patterns

Brood production patterns were evaluated to determine whether the *Wolbachia* variant wSinvictaA induces CI in *S. invicta.* Data from the two cohorts were combined to test for evidence of CI, since the shorter observation period of cohort 2 had no effect on the ability to discern anomalous brood production patterns. In the sample of uninfected individuals, three produced no viable brood, consistent with a complete FM CI phenotype, and seven produced at least one sexual larva (presumably male; sex of larvae cannot be discerned at early instars), consistent with either MD CI or DMP (Table 2).

**Table 1.**
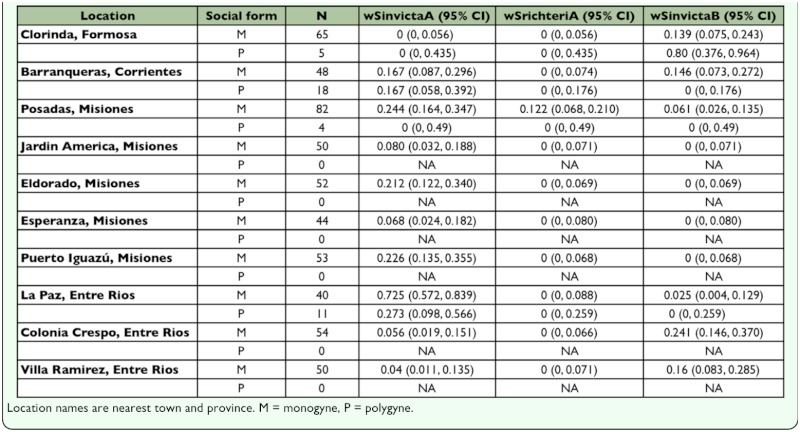
Prevalence, with 95% confidence intervals, of different *Wolbachia* variants in populations of the fire ant *Solenopsis invicta* sampled in Northeastern Argentina (May–June 2004).

The proportion of putative expressers of complete FM CI, 0.014 (95% CI = 0.005, 0.039) of the uninfected queens (3 individuals), was significantly less than the expected value of 0.161 (35 individuals; non-overlap of 95% confidence intervals) given the population wSinvictaA prevalence of 0.161 (Table 2). (Seven uninfected individuals that died before the end of the study of unknown ailments lived for at least two weeks, which allowed enough time to observe that they were infertile, however even if these individuals are conservatively included as putative FM CI expressers, the total [10 individuals; 0.044; 95% CI = 0.024, 0.079] still falls significantly short of expected.) Of the infected queens, one individual failed to rear any brood, which would not be predicted to be induced by FM CI.

**Table 2.**
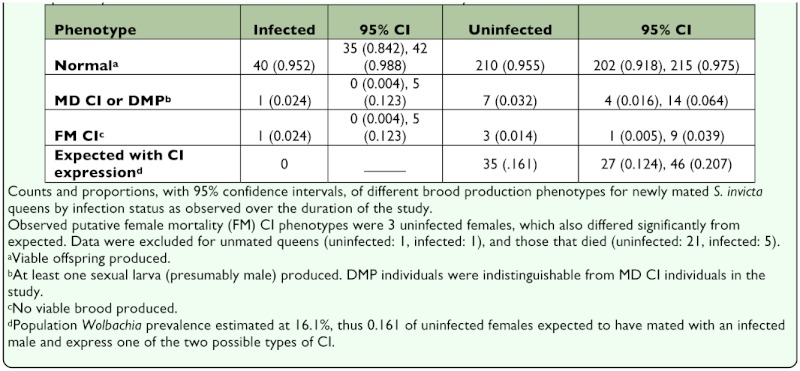
Counts and proportions, with 95% confidence intervals, of different brood production phenotypes for newly mated *S.invicta* queens by infection status as observed over the duration of the study.

**Figure 1.**
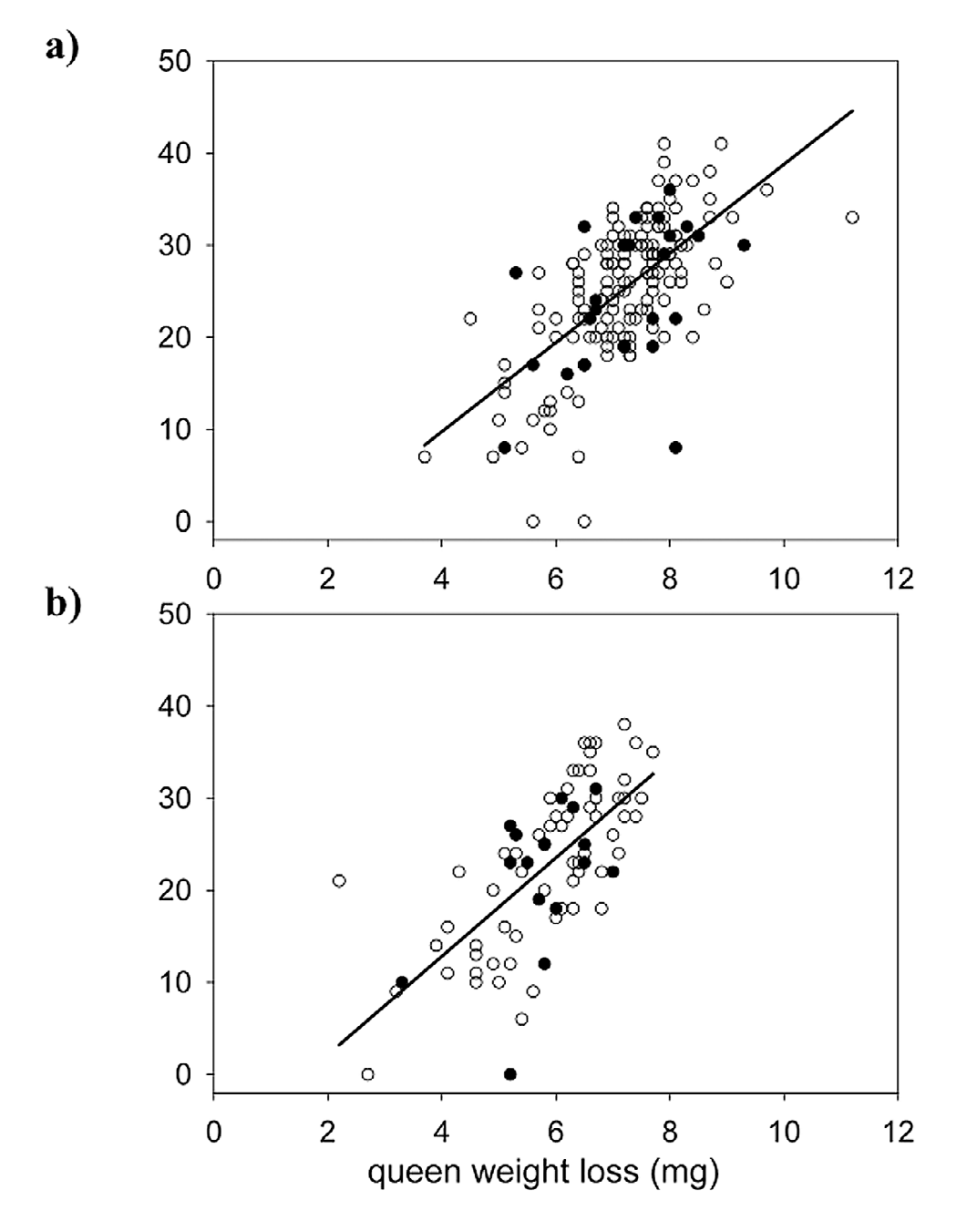
Total offspring production by newly mated queens as a function of *Solenopsis invicta* queen weight loss. *Wolbachia*-infected queens are represented by filled circles, and uninfected queens by open circles. (A) Cohort 1: Colonies collected on the day the first adult worker eclosed, or, for two queens that id not produce viable brood, at the end of the study; (B) Cohort 2: Colonies collected after 17–18 days of development. High quality figures are available online.

Another possibility is that wSinvictaA induces incomplete FM CI in *S. invicta* queens. However, visual inspection of frequency distributions of total offspring production by uninfected queens of each cohort provides no evidence for bimodality, which is expected if queens expressing strong incomplete FM CI cluster at very low productivity. Additionally, Figure 1a & b reveal a wide range in productivity for both infected and uninfected queens along the *y*-axis and no clear clustering of points for uninfected queens at very low productivity, suggesting that strong incomplete FM CI is unlikely.

The proportion of putative expressers of MD CI was 0.032 (95% CI = 0.016, 0.064), also far short of the proportion expected for a population with 16.1% infection prevalence (Table 2). An additional infected queen produced sexual larvae (presumably male). In total, production of at least one sexual larva by 3.1% of all queens (7 uninfected queens + 1 infected queen) was observed. Interestingly, for all eight of these queens, within a few days of when a larva became recognizable as a sexual larva, it was killed and eaten by its mother, and these resources might be reinvested into trophic eggs or worker brood. A likely explanation for the production of sexuals by these queens is that they were diploid-male producers, rather than queens expressing MD CI. Indeed, Ross et al. (1993) found a very similar percentage of queens collected from nearby Corrientes, Argentina producing dipliod males (3.2%).

### Queen weight and body size

No evidence for effects of *Wolbachia* infection on either queen weight (at the beginning of observations) or on queen body size was found, when measured as head width (Table 3). The 95% confidence intervals for the mean difference between treatments are shown to assess confidence in significance testing (Hoenig and Heisey 2001). Since 95% confidence intervals for the mean difference between infected and uninfected individuals for both body weight and head width were tightly bracketed around zero, these data rule out likely biologically meaningful effects of infection with high confidence (Table 3).

**Table 3.**
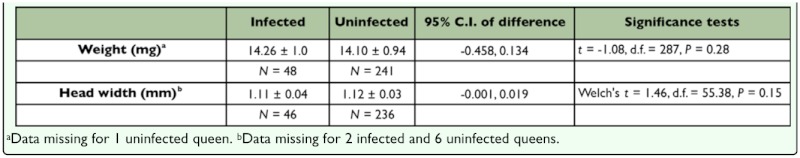
Mean values ± SD, 95% confidence intervals for the mean difference between infected and uninfected individuals, and statistical analysis of weight (mg) and head width (mm) of newly mated Solenopsis invicta queens by Wolbachia infection status.

### Survival

Due to the slight differences in protocols between cohorts 1 and 2 (i.e. when colonies were collected), all analyses of survival and productivity were conducted separately for each cohort. Survival was not significantly correlated with *Wolbachia* infection for either cohort 1 or 2 (Table 4). In cohort 1, two out of 27 infected and 13 of 169 uninfected queens died before the end of the study (7.4 vs. 7.7%, respectively). Kaplan-Meier product-limit survival estimates to day 32 (median day of first worker emergence) and to the end of the study were 0.93 (95% CI = 0.831, 0.99) for infected queens, while survival for uninfected queens was 0.93 (0.891, 0.969) to day 32 and 0.74 (0.413, 0.99) to the end of the study. The large confidence interval for survival to the end of the study for uninfected queens indicates that this survival estimate should be viewed with caution. Indeed, while survival estimates for both infected and uninfected individuals to day 32 were based on robust sample sizes, a large proportion of the queens had been collected (and censored) after day 32, thus survival estimates based on mortality events late in the study were based on small sample sizes. In cohort 2, three of 21 *Wolbachia*-infected and six of 71 uninfected queens died (14.3 vs. 8.5%, respectively). Kaplan-Meier product-limit survival estimates to the end of the study (day 18) for infected queens were 0.86 (0.71, 0.99), and 0.92 (0.855, 0.985) for uninfected queens.

### Queen productivity-cohort 1

Offspring production of all stages (adults, pupae, and larvae) by uninfected queens was not significantly different from *Wolbachia*-infected queens (Table 4). Ninety-five percent confidence intervals for the mean difference in productivity between infected and uninfected queens include zero, but because of high variance in these data, they also include both positive and negative values that could be biologically meaningful (in the case of total offspring, up to a 11% increase or 16% decrease). Also, significant effects of *Wolbachia* infection on the developmental time of offspring (days to eclosion of the first adult) or on the amount of weight lost by queens during the pre-emergence period were not observed (Table 4). In both cases the data were less variable than for productivity, with 95% confidence intervals for the difference between infected and uninfected individuals allowing up to a 2.7% reduction or a 1.8% increase for emergence time, and up to a 5.6% increase to 6.5% decrease for weight loss. During claustral colony founding, queens rear the first generation of workers on trophic eggs and salivary secretions produced solely from their own fat stores, and previous studies have shown that productivity is correlated with the amount of weight they lose during the claustral period (Tschinkel 1993). Multiple regression was used to determine whether *Wolbachia* infection affected the efficiency with which queens converted their fat stores into offspring. As expected, regression of counts of total brood (B_total_) on queen weight loss (L) yielded a significant model (Figure 1A; Table 5). A larger negative *y*-intercept or shallower slope for infected queens versus uninfected queens would indicate that reduced efficiency was associated with infection. However, a class variable for infection status (I) and an interaction term (L × I), were not significant in the model, indicating that *Wolbachia* infection had no effect on either the *y*-intercept (queen productivity at zero weight loss) or the slope of the model (rate at which queens converted fat stores into brood numbers).

**Table 4.**
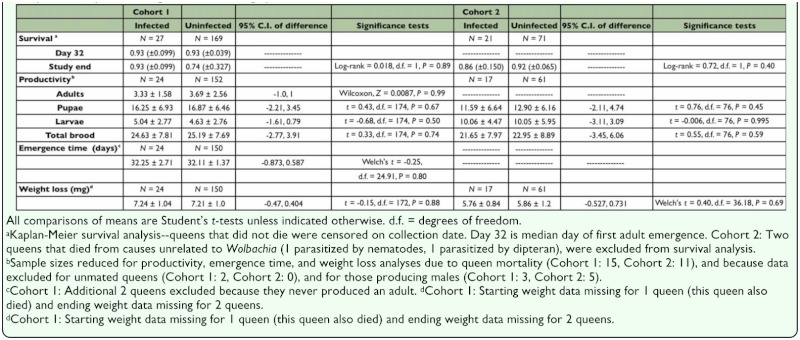
Mean values ± SD and statistical analysis of productivity, emergence time, weight loss, and survival (± 95% confidence interval) of newly mated *Solenopsis invicta* queens during claustral founding by *Wolbachia* infection status.

### Queen productivity-cohort 2

Although the experimental protocol was slightly different between the two cohorts, the qualitative and quantitative results obtained for cohort 2 were similar to those from cohort 1. There was no significant effect of infection on brood production (Table 4). As for cohort 1, because of high variance in the data, confidence intervals for the difference between infected and uninfected individuals for total brood production include zero, but do not rule out potentially biologically meaningful effects (+15% to -26%; Table 4). Also, the mean total offspring production of cohort 2 differed by less than three individuals from that of cohort 1, indicating that the shorter observation period of cohort 2 was a close approximation of the entire pre-emergence period (Table 4). As for cohort 1, regression of counts of total brood (B_total_) on queen weight loss (L) was significant (Figure 1B; Table 5), but again, infection status (I) and weight loss by infection (L × I) were not significant.

### *Wolbachia* transmission fidelity to virgin queens and males

All 190 individual alate queens assayed from 26 monogyne families (median sample per family = 7.0) with wSinvictaA infections were positive for this variant (95% CI = 0.98, 1), demonstrating that the fidelity of wSinvictaA transmission is near-perfect to perfect. In addition, all 105 males assayed from 13 families (median sample per family = 6.0) known to harbor wSinvictaA were infected (95% CI = 0.97, 1), indicating that males readily survive wSinvictaA infections.

**Table 5.**
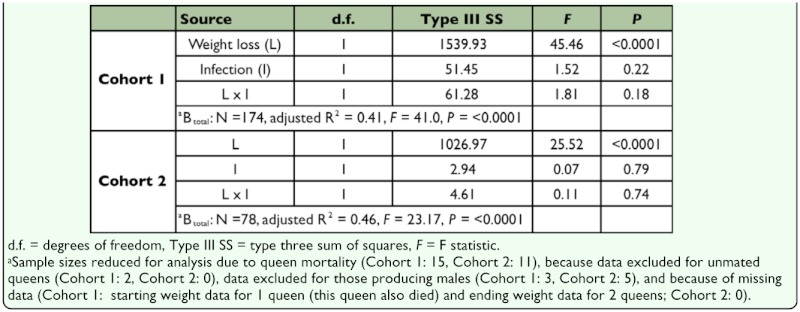
Total offspring produced (Btotal) by newly mated *Solenopsis invicta* queens during simulated colony founding as a function of weight loss during the claustral period (L), *Wolbachia* infection status (I), and L by I in multiple regression. Separate models were fitted for Cohort 1 and Cohort 2.

## Discussion

The aim of the present study was to determine the phenotypic effects induced by a *Wolbachia* variant, wSinvictaA, in its natural fire ant host *Solenopsis invicta.* To achieve this objective, brood production patterns were studied for evidence of the expression of cytoplasmic incompatibility (CI), and fitness correlates of newly mated queens were measured during the claustral stage of colony founding for evidence of fitness effects. In addition, *Wolbachia* prevalence in host populations and transmission fidelity to male and female reproductive offspring in mature colonies in the field were measured. Below the findings are discussed in relation to potential CI, sex-ratio distortion, and fitness effects of this *Wolbachia* variant in *S. invicta*.

### Cytoplasmic incompatibility

The results provide no clear evidence for *Wolbachia-*induced CI. It was possible to rule out male development (MD) CI as a phenotype for wSinvictaA in *S. invicta,* since significantly less male production than expected was observed and that which was observed most likely represents diploid male production. In addition, cumulative results suggest that female mortality (FM) CI is unlikely. A phenotype of complete FM CI alone can be ruled out since far fewer infertile queens were observed than expected. However, because studies of FM CI in the field have observed a range in expression (depending upon male age and other factors; reviewed by Hoffmann and Turelli 1997), the possibility that the three infertile uninfected individuals observed in the sample were expressing complete FM CI, and the possibility that the remaining CI-expressing individuals (expected number equals 32 individuals) were cryptic in the dataset cannot be entirely ruled out because they expressed incomplete FM CI.

The survey data suggest that it is unlikely that this variant induces incomplete FM CL Of the eight new populations identified with wSinvictaA in this study, only one had a prevalence above the theoretical minimum stable prevalence for CI-*Wolbachia* of 50% (Hoffmann and Turelli 1997), bringing the total to 12 of 13 surveyed populations with wSinvictaA below 50%. It should be noted that the possibility that the low prevalence of infections results from very recent invasion of *Wolbachia* into fire ant populations such that infections are still in the process of sweeping through these populations is not plausible either since a previous study demonstrated that wSinvicta A infections are likely to be ancient in *S. invicta* (Ahrens and Shoemaker 2005).

A final speculative scenario is that the wSinvictaA variant does induce incomplete FM CI, but the vertical transmission and fitness effects of this variant are such that the predicted unstable equilibrium prevalence (invasion threshold) is so high that it is unlikely to ever be reached by chance alone (Hoffmann and Turelli 1997). Under this scenario, infections could be maintained locally despite frequent infection loss from individual populations by repeated invasions of wSinvictaA into *S. invicta* populations via horizontal transmission. However, using the models of Hoffman and Turelli (1997), the data suggest this scenario for incomplete FM CI is unlikely as well: assuming a negative fitness cost of 16% associated with wSinvictaA infections (the upper confidence limit from the productivity tests), near perfect transmission (0.981, the lower 95% confidence interval for virgin queens), and complete CI, the stable equilibrium frequency of wSinvictaA, would be 1.0 and the invasion threshold would be 0.17. Lowering the fitness cost or segregation rate lowers the invasion threshold frequency while reducing the strength of CI raises this invasion threshold, but this is likely to be of little consequence since even small CI effects should eliminate colonies under high competition with non-expressing con-specifics (see below: Van Borm et al. 2001; Wenseleers et al. 1998). Thus, the fact that a prevalence greater than 0.17 was observed in several populations (including Puerto Iguazú) does not support the hypothesis that negative fitness effects and imperfect transmission are sufficient to prevent a CI-inducing wSinvictaA from reaching the invasion threshold frequency. In summary, these data suggest that the possibility the wSinvictaA variant induces MD or FM CI in *S. invicta* is remote.

### Sex-ratio distortion

The low observed prevalence of wSinvictaA at Puerto Iguazú and other sites is consistent with patterns expected for some sex-ratio distorting *Wolbachia* variants. For example, *Wolbachia* variants that kill infected host males commonly persist at low equilibrium prevalence (1–30%) such as we observed for wSinvictaA (Hurst et al. 1997). However, unlike most hosts infected with MK *Wolbachia* (Hurst et al. 1997), *Wolbachia-*infected *S. invicta* males were easy to find, and wSinvictaA infections were detected in every male surveyed from infected *S. invicta* colonies. Thus, it is clear that males readily survive wSinvictaA infections, suggesting that if wSinvictaA were a male-killer there would have to be very high host resistance to male-killing, with only a fraction of males dying. Although unlikely, completely ruling out such an effect would require measuring sex-ratios in a large number of mature colonies by infection, which was not feasible in the current study. Additional lines of evidence suggest that two other described *Wolbachia* sex-ratio-distorting phenotypes, feminization and parthenogenesis (Rigaud 1997, Stouthamer 1997), are improbable. For example, the finding that many males are infected argues against the possibility of feminizing *Wolbachia,* which convert infected males to females. Also, parthenogenesis can be ruled out because the genotype distributions among offspring from monogyne (single-queen) colonies headed by wSinvictaA-infected queens are fully consistent with all offspring being produced via sexual reproduction (single female mated to single male; D.D.S. unpublished data).

### Fitness correlates

The possibility of direct fitness effects was also considered, including direct positive fitness effects since these could be important for the maintenance of infections in populations in the absence of CI or sex-ratio distortion. The claustral colony founding period is a highly appropriate stage in the colony cycle to examine for direct fitness effects since it is a very stressful stage for queens (Tschinkel 1992, 1993) where any positive *Wolbachia* effects would likely be most beneficial and where it would likely be most difficult for the host to compensate for negative effects (Brown et al. 2000, 2003). Studies have shown that the most important queen phenotypic trait for success during early pleometrotic colony founding is relative fighting ability in queen contests (Bernasconi and Keller 1999), the best predictor of which is relative queen head width (a correlate of body size). We tested for a correlation between *Wolbachia* infection status and head width (*Wolbachia* have been shown to be associated with reduced body size in *Drosophila* (Hoffmann et al. 1998)). Queen weight was also measured soon after collection from mating flights since queen weight is primarily a measure of fat stores and has been shown to be correlated with productivity (but see DeHeer 2002; Tschinkel 1993). Queen weight is largely dependent upon the natal colony (Keller and Passera 1989), and probably best viewed as a measure of the condition of the natal colony. Finally, whether *Wolbachia* infections affect the ability of queens to convert their energy stores into offspring during simulated claustral founding was examined, since the quantity of workers produced by either pleometrotic or haplometrotic queens is critical to the success of incipient colonies. Mortality of young colonies often is caused by loss of brood to con-specific brood raiding (Adams and Tschinkel 1995), and colonies with more workers are more successful in brood raiding if challenged by another incipient fire ant colony (Tschinkel 1992).

The main results were that the wSinvictaA *Wolbachia* variant had no detectable effects on queen size (measured as head width) or weight, the quantity of offspring produced, the amount of queen weight lost prior to emergence of workers, the efficiency with which queen weight is converted into offspring numbers, queen survival, or the time needed for brood to develop into adults. The lack of any discernable fitness effects coupled with lack of evidence for any phenotypic effects raises the issue of whether this variant is anything more than a passive passenger in fire ants and leave open the question as to what factors explain how the wSinvictaA variant is maintained in *S. invicta* populations. Below several possibilities are discussed that take into account the combined results as well as those from previous studies.

Other *Wolbachia* variants that do not appear to induce CI and occur at very low prevalence have been described in *Drosophila simulans* (Hoffmann et al. 1996) and *D. yakuba* (Charlat et al. 2004), similar to wSinvictaA in *S. invicta.* Theoretical models (Hoffmann and Turelli 1997) predict that such non-CI inducing *Wolbachia* infections can be maintained in host populations if maternal transmission is perfect, which appears to be the case for non-CI inducing *Wolbachia* infecting *D. simulans* and *D. yakuba* (Charlat et al. 2004; Hoffmann et al. 1996) or if these variants induce positive fitness effects. Charlat et al. (2004) also hypothesized that if maternal transmission fidelity was not perfect (their 95% CI allowed for up to 0.02 segregation), then a non-CI *Wolbachia* could persist in the absence of positive fitness effects as long as the rate of loss of infections took place at roughly the same rate as horizontal transmission replaced them.

Because maternal transmission fidelity of wSinvictaA in *S. invicta* was found to be very close to being perfect, a low rate of horizontal transmission potentially could allow *Wolbachia* infections to persist at low prevalences in Puerto Iguazú and elsewhere. In fact, there is evidence for frequent horizontal transmission of *Wolbachia* in *S. invicta* at least on an evolutionary time scale. Ahrens and Shoemaker (2005) concluded that the phylogenetic history of wSinvictaA infections in *S. invicta* has involved multiple invasions or horizontal transmission events, and Dedeine et al. (2005) presented phylogenetic evidence for horizontal transmission of *Wolbachia* between *S. invicta* and a social parasite. Whether such seemingly rare events are sufficient to maintain *Wolbachia* in host populations depends upon the transmission fidelity of wSinvictaA being very high. While no loss of wSinvictaA infection (segregation) was observed in the surveys of virgin queens from mature *S. invicta* infected colonies, the lower 95% confidence interval allows for a segregation rate of up to 0.02. Importantly, phylogenetic studies of *S. invicta* and its *Wolbachia* variants by Ahrens and Shoemaker (2005) have shown that wSinvictaA transmission are clearly not perfect since infections have been repeatedly lost from host mtDNA lineages over time.

On the other hand, if the segregation rate is in reality closer to 0.02 than zero, then it seems plausible that some positive fitness effects are needed in order to maintain infections in host populations. For example, assuming a segregation rate of 0.02, Hoffman and Turelli's (1997) models predict that small positive effects of 2.1 to 2.7% can allow infections to persist in the range of prevalence that was observed (a larger positive effect of approximately 7.7% is required for La Paz, where the prevalence of the wSinvinctaA variant is 0.72 ). One noteworthy point here is that although clear evidence for positive effects in analyses of queen productivity were not observed, only large fitness effects of the magnitude observed in some other *Wolbachia* studies (Dobson et al. 2002 Weeks et al. 2007) likely would have been detectable given the high variance in queen productivity: small positive fitness effects of the wSinvictaA variant may exist, but simply were not detected. Indeed, because analyses of fecundity would not have detected positive effects less than 10%, fitness increases of 2.1 to 2.7% are well within the realm of possibility. Moreover, it is also possible that the wSinvictaA variant affects fire ant fitness in ways that were outside the scope of the present study (e.g. conferring resistance to parasitoids (Hsiao 1996) or having effects occurring later in the colony cycle). Nonetheless, the finding that initial queen weight did not differ between infected and uninfected queens suggests at the very least the natal colonies of these queens were in similar condition (Keller and Passera 1989).

### *Wolbachia* in New World ants

Our results add to recent studies of the *Wolbachia* infecting New World ants, nearly all of which belong to a single groups (or previously two groups, *InvA* and *InvB,* based solely on *wsp* sequences) (Russell et al. 2009). The remarkable similarity among *Wolbachia* variants infecting New World ants, and the fact that these variants are found only in ant hosts some of which are both phylogenetically and geographically disparate, has led several authors to speculate that these *Wolbachia* variants are ant-adapted specialists that have been frequently horizontally transmitted among ant species (Dedeine et al. 2005; Reuter et al. 2005; Russell et al. 2009; Tsutsui et al. 2003; Van Borm et al. 2003). However, little is known about what phenotypic effects these putative ant specialists induce in their hosts or even what selection on *Wolbachia* in symbiosis with ants may entail (reviewed by Zientz et al. 2005). In one of the few other studies examining phenotypic effects of these *Wolbachia,* Van Borm et al. (2001) found infections to be less prevalent in workers and males than in queens of two species of leafcutter ants (*Acromyrmex echinatior* and *A. octospinosus*) in Panama. Van Borm et al. (2001) concluded that the lower prevalence of infections in males was consistent with a male-killing phenotype. Van Borm et al. (2001) also speculated that the lower prevalence of infections in workers is consistent with clearance of infection, which potentially could boost host colony productivity assuming there is a physiological cost of infections (see also Wenseleers et al. 2002). While wSinvictaA is very similar to the two variants infecting *Acromyrmex,* based on *wsp* and 16S sequences, the data indicate that its life history and host effects probably are quite different suggesting that adaptation to ant hosts does not limit *Wolbachia* to one particular phenotype, but instead may induce very different phenotypic effects. Further research is needed on other *Wolbachia* variants in *S. invicta* to determine the extent of diversity of *Wolbachia* phenotypic effects on fire ants and provide insight into the nature of selection on ant *Wolbachia*.

## Abbreviations

**CI**,: cytoplasmic incompatibility;
**FM**,: female mortality; **M**, monogyne;
**MD**,: male development;
**MK**,: male-killing;
**mtDNA**,: mitochondrial DNA;
**P**,: polygyne
